# Transcriptome sequencing of *Eucalyptus camaldulensis* seedlings subjected to water stress

**DOI:** 10.1186/1753-6561-5-S7-O31

**Published:** 2011-09-13

**Authors:** Bala Thumma

**Affiliations:** 1 CSIRO Plant Industry, Australia

## 

Water stress limits plant survival and production in many parts of the world. Identification of genes responding to water stress conditions will underpin efforts to breed plants better adapted to drought. We studied the effect of water stress on *Eucalyptus camaldulensis* seedlings derived from three natural populations. Physiological and growth traits were measured and gene and allelic expression in leaves was examined by RNA sequencing (RNA-seq). Water stress had a significant impact on all the physiological and growth traits, while differences between the populations were not significant. Genes differentially expressed in leaves were identified by *de novo* assembly and by *ab initio* transcriptome mapping using the *Eucalyptus grandis* reference genome sequence. Gene ontology (GO) enrichment tests with 2,500 significantly differentiated genes revealed 128 stress-related gene categories were up-regulated while 28 gene categories belonging to photosynthesis and other metabolic processes were down-regulated under stress treatment. More than 190,000 single nucleotide polymorphisms (SNPs) and small indels were detected and 4,053 of these revealed differential allelic expression between control and drought stressed seedlings. Allelic expression of 70% of these variants was correlated with total gene expression. These variants may be *cis*-acting variants or in high linkage disequilibrium with such variants. The SNPs and indels identified in this study form a useful resource for further testing in association studies.

